# Peripheral Nerve Blockade in Total Hip Arthroplasty: A Retrospective Study with Propensity Score Matching

**DOI:** 10.3390/jcm12175514

**Published:** 2023-08-25

**Authors:** Hyuck Min Kwon, Tae Sung Lee, Heon Jung Park, Bora Lee, Yong Seon Choi, Kwan Kyu Park

**Affiliations:** 1 Department of Orthopedic Surgery, Severance Hospital, Yonsei University College of Medicine, Seoul 03722, Republic of Korea; hyuck7777@yuhs.ac (H.M.K.); skisports88@yuhs.ac (T.S.L.); ryan0912@yuhs.ac (H.J.P.); 2 Department of Anesthesiology and Pain Medicine, Severance Hospital, Yonsei University College of Medicine, Seoul 03722, Republic of Korea; dreamkaist@yuhs.ac

**Keywords:** total hip arthroplasty, peripheral nerve block, leg lengthening

## Abstract

The effect of peripheral nerve block (PNB) according to leg lengthening following total hip arthroplasty (THA) has not been studied yet. The purpose of this study was to investigate the effect of PNB according to the change in leg length after THA. From January 2016 to August 2021, 353 patients who underwent unilateral THA for osteonecrosis of the femoral head or osteoarthritis of the hip joint were retrospectively reviewed. The patients were divided into two groups for comparison: 217 patients who controlled postoperative pain using only intravenous venous patient-controlled analgesia (IV PCA) (PCA group) and 136 patients who controlled postoperative pain using PNB and IV PCA (PCA + PNB group). We further divided the patients into two groups (leg lengthening after surgery < 10 mm and >10 mm) and compared them. After propensity score matching, the PCA and PCA + PNB groups, with 134 patients each, were compared and analyzed. The pain intensity at rest was significantly lower in the PCA + PNB group compared with that in the PCA group at postoperative 6, 24, and 48 h (*p* = 0.0001, 0.0009, and <0.0001, respectively). In the subgroup analysis, for patients whose limb lengthening was less than 10 mm after THA, the pain intensity at rest was significantly lower in the PCA + PNB group compared with that in the PCA group at postoperative 24 and 48 h (*p* = 0.0165 and 0.0015, respectively). However, in patients whose limb lengthening was more than 10 mm after THA, there was no significant difference between the pain intensity at activity and rest in the two groups at postoperative 6, 24, and 48 h (*p* > 0.05). PNB did not show superiority in terms of pain reduction in patients whose limb lengthening was more than 10 mm after THA. Further investigations on methods for reducing pain in patients whose leg length is increased by more than 10 mm are needed.

## 1. Introduction

Restoring the normal hip biomechanics in total hip arthroplasty (THA) is the most important factor to obtain excellent surgical outcomes and functional recovery [1,2,3,4]. In particular, leg length discrepancy (LLD) after THA could be directly related with patient dissatisfaction due to limping gait, gait disorder, and back and leg pain [5,6]. Therefore, the assessment of leg length before surgery and restoring of leg length in THA should be a priority. In most patients requiring THA, the more progressed the disease, the shorter the leg length is due to loss of cartilage and bone or soft tissue contracture. As such, the leg length is generally restored by appropriately increasing it during THA [7,8,9]. However, larger leg lengthening following THA could induce smaller hip flexion range of motion, since it leads to increased soft tissue tension. In addition, leg lengthening in THA can cause injury to the sciatic and femoral nerves, which can cause symptoms related to nerve palsy, often leading to neuropathic pain [10,11,12]. Therefore, some studies have discussed the importance of intraoperative electromyography or postoperative electromyography examination to diagnose and prevent nerve damage after THA [10,13,14].

Several studies have shown that peripheral nerve block (PNB) after THA has excellent advantages in terms of pain control, as well as in decreasing the risk of various postoperative complications in patients [15,16,17]. There are many methods of PNB that can be applied to THA. Mostly, femoral nerve block, quadratus lumborum block, fascia iliaca compartment block, lateral femoral cutaneous nerve block, and pericapsular nerve group block after THA are performed using ropivacaine or bupivacaine under ultra-sound guidance. Since the leg lengthening in THA can result in significant postoperative pain due to more soft tissue release and nerve stretching [18], PNB can be expected to have a better effect after THA with leg lengthening, considering its great effect in reducing acute postoperative pain. However, the effect of PNB according to limb lengthening following THA has not been studied yet.

We hypothesized that the increased leg lengthening during THA could cause more postoperative pain after surgery. As such, it was assumed that the effect of PNB would be better for patients with significant leg lengthening. The purpose of this study was to investigate the effect of peripheral nerve block according to the change in leg length after THA.

## 2. Materials and Methods

### 2.1. Study Population

After Institutional Review Board approval was obtained, electrical medical records were retrospectively reviewed. We collected data from 544 patients who consecutively underwent unilateral THA performed by a senior experienced orthopedic surgeon in a tertiary hospital from January 2016 to August 2021. Patients who had hip fracture, inflammatory hip arthritis, rheumatoid arthritis, any infection history of hip joint, revision surgery, and patients who need special devices due to severe instability, anatomical deformity, and bone defects were excluded. Patients requiring excessive soft tissue release due to an LLD > 30 mm were also excluded. Finally, among the 544 patients, 353 patients who underwent unilateral THA for osteonecrosis of femoral head or osteoarthritis of the hip joint were enrolled in this study. The patients were divided into two groups: 217 patients who controlled postoperative pain using only intravenous venous patient-controlled analgesia (IV PCA) (PCA group), and 136 patients who controlled postoperative pain using PNB and IV PCA (PCA + PNB group). Propensity score matching was performed for age, sex, body mass index, American Society of Anesthesiologists score, and diagnosis among these groups. Furthermore, demographic data, radiologic data, and clinical outcomes were assessed in all patients.

### 2.2. Surgical Procedure

All the THA procedures were performed by a single surgeon (KKP) with the patient in a lateral decubitus position and stabilized with the aid of a pelvic positioner. All the surgeries were performed using a posterolateral hip approach and the short external rotators were repaired. Cementless acetabular cups and tapered wedge stems were used in all cases. All the cases used ceramic liner and ceramic head. In all cases, hip stability was assessed after final acetabular cups, liner, and final femoral broach with trial head and neck component were implanted. To minimize LLD, preoperative template method was routinely conducted using standard hip radiographs with magnification markers. The femoral component size and osteotomy level were determined. Intraoperatively, the uninvolved lower limb was used as a reference, considering the relative difference at the patella in the lateral decubitus position [19]. To optimize soft tissue tension, the neck option was selected according to the push–pull test (Shuck test).

### 2.3. Postoperative Pain Management

Thirty minutes before the end of the surgery, IV fentanyl 1 µg/kg and palonosetron 0.075 mg were administered to the patient for postoperative analgesia and antiemetic effects, respectively. All the patients were administered IV PCA for 48 h postoperatively, which comprised fentanyl 7 µg/kg and palonosetron 0.075 mg (total volume including saline: 100 mL), delivered as a 2 mL/h background infusion and 0.5 mL doses on patient demand, with a 15 min lockout time. In the ward, all the patients were administered celecoxib 200 mg orally and acetaminophen 1 g intravenously every 12 h. All the patients started postoperative exercise following the same rehabilitation protocol. Alongside this, bedside exercises (ankle pumps, quadriceps stretching, leg raising) were performed 0–6 h after the operation. Standing with a walker ambulation was permitted on postoperative day 1 following the same protocol for all patients. Additional pain control was administered as rescue medication (pethidine 25 mg, pethidine 50 mg, tramadol 50 mg) through intramuscular injections.

### 2.4. Radiologic Assessment

Preoperative and postoperative LLD of the hips of the patients were measured in full-length antero-posterior standing radiographs on the day before surgery and 3 months after surgery, respectively. As landmarks for LLD measurement, bilateral radiologic teardrops at the pelvis and the centers of the lesser trochanters at the femurs were used. The distance between these two landmarks was measured in terms of the change in leg length before and after THA. The degree of measurement reliability was evaluated using intraclass correlation coefficients (ICC). Calculation of the ICC was performed by two experienced orthopedic surgeons (HMK, JYP). For the ICC, values less than 0.2 were considered to indicate poor agreement; 0.21 to 0.40, fair agreement; 0.41 to 0.60, moderate agreement; 0.61 to 0.80, good agreement; and above 0.80, excellent agreement [20].

### 2.5. Clinical Outcome Assessment

Clinical outcome assessment was performed before surgery and 3 months after using the Harris Hip Score and Western Ontario and McMaster Universities Osteoarthritis index. The pain intensity at rest and during activity was evaluated using an 11-point numeric rating scale (0 = no pain, 10 = worst imaginable pain). Pain scores were assessed at three-time points, 6, 24, and 48 h after surgery.

### 2.6. Statistical Analysis

Descriptive statistics were performed, and normality distribution was assessed by the Shapiro–Wilk test. Continuous variables were analyzed using the Student’s *t*-test (normal distributions) or Mann–Whitney test (non-normal distributions). Categorical variables were compared using the chi-square test or Fisher’s exact test. *p*-value < 0.05 was considered statistically significant in all cases. After propensity score matching, continuous variables were analyzed using Wilcoxon signed rank test, and categorical variables were analyzed using the McNemnar’s test. The analysis of data and propensity score matching were performed using the SAS software (version 9.4, SAS Inc., Cary, NC, USA).

## 3. Results

After propensity score matching, two groups of 134 patients each were compared and analyzed. The baseline characteristic of the patients, diagnosis, preoperative LLD, and limb lengthening after surgery are presented in Table 1. There were no statistically significant differences between the groups in the preoperative LLD (4.1 mm vs. 3.3 mm, *p* = 0.7000) and limb lengthening after surgery (4.9 mm vs. 3.7 mm, *p* = 0.0616).

Table 2 shows the preoperative and postoperative clinical scores. The pain intensity at rest was significantly lower in the PCA + PNB group compared with that in the PCA group at postoperative 6, 24, and 48 h (*p* = 0.0001, 0.0009, and <0.0001, respectively). However, there was no significant difference in the pain intensity at activity between the two groups at postoperative 6, 24, and 48 h (*p* > 0.05). In addition, there was no significant difference between the two groups in terms of preoperative and postoperative clinical scores.

We further divided the patients into two groups according to leg lengthening (<10 mm and >10 mm) and compared them. After propensity score matching, the pain intensity at rest in postoperative 6 h after surgery in the group with more than 10 mm of leg lengthening was higher than that in the group with less than 10 mm of leg lengthening. There were no statistically significant differences between groups in terms of pain intensity at rest and activity (Table 3).

Propensity score matching of the 190 patients with leg lengthening < 10 mm after surgery allowed comparing 42 patients from the PCA + PNB group to 42 patients from PCA group. The pain intensity at rest was significantly lower in the PCA + PNB group compared with that in the PCA group at postoperative 24 and 48 h (*p* = 0.0165 and 0.0015, respectively). However, there was no significant difference in pain intensity at activity between the two groups at postoperative 6, 24, and 48 h (*p* > 0.05) (Table 4).

Propensity score matching of 163 patients with leg lengthening > 10 mm was performed after comparing 26 patients from the PCA + PNB group to 26 patients from PCA group. There was no significant difference in pain intensity at activity and rest between the two groups at postoperative 6, 24, and 48 h (*p* > 0.05) (Table 5).

For all patients, we calculated the total count of rescue medication prescribed during the first two postoperative days, converting the total dose of rescue medication into a total morphine equivalent dose, in mg, using converting factors [21]. The average count of opioids consumed during the first two postoperative days decreased by 0.35 when a PNB was used in addition to PCA, decreasing by a morphine equivalent of 2.8 mg; however, these results were not statistically significant. Even after subgroup analysis, there was no significant difference in total morphine equivalent dose according to the leg lengthening.

## 4. Discussion

This study investigated the analgesic effect and early postoperative functional outcomes of PNB according to change in leg length after THA. The principal finding of this study was that immediate postoperative pain intensity in the first 48 h of THA was significantly lower in PCA with PNB than in PCA alone. In particular, in the subgroup analysis according to leg lengthening < 10 mm or > 10 mm, there was no statistical difference in the pain reduction effect of PCA with PNB compared to the PCA alone. In addition, the effect of PNB on rescue medication was not affected by the difference in leg lengthening after THA. It can be assumed that the pain relief effect of PNB was greater in patients whose leg lengthening after THA was less than 10 mm.

The pain relief effect of PNB in THA at early postoperative period is well known, and PNB has been considered an essential component of pain management [15,22,23,24]. In this study, femoral nerve block, quadratus lumborum block, fascia iliaca compartment block, lateral femoral cutaneous nerve block, and pericapsular nerve group block after THA were performed according to the decision of the anesthesiologist in our institution. Although PNB showed a good overall effect in reducing pain at rest up to 48 h after THA in this study, it was not superior when only patients with a leg lengthening > 10 mm were considered. Our results show that PNB, which blocks the nerve by injection, is less effective in patients who need more than 10 mm of leg lengthening.

In the case of hip diseases requiring THA, shortened leg length can be found in many cases, and proper leg length restoration is very important for obtaining excellent surgical outcome [25]. Although the good analgesic effect of PNB in THA is well known, to our knowledge, the association with the degree of limb lengthening after THA has not been studied yet. In this study, we demonstrated that following THA early postoperative resting pain could be higher in cases of leg lengthening > 10 mm compared to cases of leg lengthening < 10 mm. However, since the effects of PNB were not significant after THA with a leg lengthening >10 mm, additional pain management methods would be needed. In particular, changes in leg length of more than 10 mm after THA are not only simple changes in leg length, but also complicated changes in hip joint biomechanics due to changes in the center of the hip joint and accompanying changes in the relative position of the pelvis and spine. This means that as the patient starts walking after THA, the patient may feel pain with a greater change in biomechanics. Therefore, patients with a leg length change of more than 10 mm after THA are thought to have less effect of PNB after THA.

Benedetti et al. investigated that leg lengthening of up to 20 mm after THA did not significantly alter the symmetry of hip movement in hip kinematics [26]. We confirmed that there was no difference in the functional score at 3 months after surgery according to leg lengthening > 10 mm or < 10 mm after THA. Kawai et al. demonstrated that a larger limb lengthening after THA was associated with a smaller hip range of motion because of muscular contracture and increased soft tissue tension [1]. Although there may be no differences in daily functional activities, there might be differences in deep bending activities, such as picking up an object while sitting on a chair or squatting on the floor. Longer follow-up periods may display differences in functional scores, so long-term follow-up is necessary.

Leg lengthening after THA is often accompanied by soft tissue release such as capsule, tendon, and muscle, which can then cause an increase in soft tissue tension and nerve stretching. Therefore, longer leg lengthening after THA can cause more postoperative pain. Also, the hip capsule contains a moderate and diffuse density of nociceptive fibers; therefore, the patients with leg lengthening >10 mm after surgery could feel more pain than the patients with little change in leg length. However, the hip is diffusely innervated by branches of the femoral, sciatic, obturator, and gluteal nerves, and perforating muscular branches, which hinder the control of postoperative pain with a single PNB. Therefore, in case of THA requiring leg lengthening > 10 mm, considered to require more soft tissue procedures, other methods besides PNB, such as intraoperative periarticular injection, should be applied to achieve good results in reducing postoperative pain.

This study has several limitations. First, this study was a single-center, retrospective comparative study. Since this was not a randomized controlled study, selection bias could exist. To minimize the bias, propensity score matching was performed to analyze the pain reduction effect of PNB according to leg lengthening after THA. Second, the results of this study were primarily derived from the patient’s subjective pain scale. The bias from the pain scale and survey cannot be overlooked, as it is a subjective index. Third, the information on the total amount of fentanyl could not be accurately obtained because some patients discontinued IV PCA due to side effects of it. Fourth, the different types of THA implants by manufacturers and changes in medial offset after THA can also affect postoperative pain, but we did not consider these aspects, so this is a limitation of the current study.

## 5. Conclusions

In conclusion, PNB has overall beneficial effects for early postoperative pain relief after THA. However, PNB did not show superiority in terms of pain reduction in patients whose leg lengthening was >10 mm after THA. Further investigations of methods for reducing pain in patients whose leg length is increased by more than 10 mm are needed.

## Figures and Tables

**Table 1:** Demographic data before and after propensity score matching.

	Before Propensity Score Matching			After Propensity Score Matching		
	PCA + PNB Group (N = 136)	PCA Group (N = 217)	*p* Value	PCA + PNB Group (N = 134)	PCA Group (N = 134)	*p* Value
Age (years)	61	60	0.8441	61	60	0.6438
Female, *n* (%)	77 (56.6%)	123 (56.7%)	0.9905	75 (56%)	79 (59%)	0.6276
BMI (kg/m^2^)	25.01	24.15	0.0775	24.99	25.07	0.8826
ASA			0.0561			0.7947
1	8 (5.9%)	24 (11.1%)		8 (6%)	10 (7.5%)	
2	73 (53.7%)	91 (41.9%)		71 (53%)	70 (52.2%)	
3,4	55 (40.4%)	102 (47%)		55 (41%)	54 (40.3%)	
Diagnosis			0.4006			0.8750
OA	43 (31.6%)	56 (25.8%)		42 (31.3%)	41 (30.6%)	
Secondary OA	23 (16.9%)	46 (21.2%)		23 (17.2%)	25 (18.7%)	
Osteonecrosis	70 (51.5%)	115 (53%)		69 (51.5%)	68 (50.8%)	
Preoperative LLD (mm)	−4.08	−3.95	0.5813	−4.08	−3.33	0.7000
Limb lengthening after surgery (mm)	4.90	4.03	0.1562	4.85	3.69	0.0616

Median (Q1, Q3). PCA, patient-controlled analgesia; PCA + PNB, patient-controlled analgesia and peripheral nerve block; BMI, body mass index; ASA, American Society of Anesthesiologists score; OA, osteoarthritis; LLD, leg length discrepancy.

**Table 2:** Clinical outcomes before and after propensity score matching method.

	Before Propensity Score Matching			After Propensity Score Matching		
	PCA + PNB Group (N = 136)	PCA Group (N = 217)	*p* Value	PCA + PNB Group (N = 134)	PCA Group (N = 134)	*p* Value
VAS						
0–6 h after surgery at rest	4 (2, 6)	6 (4, 7)	<0.0001	4 (2, 6)	6 (4, 7)	0.0001
0–6 h after surgery at activity	7 (6, 8)	8 (6, 8)	0.0179	7 (6, 8)	8 (6, 8)	0.2919
6–24 h after surgery at rest	2 (0, 4)	3 (2, 5)	0.0004	2.5 (0, 4)	3 (2, 5)	0.0009
6–24 h after surgery at activity	6 (5, 7)	6 (4, 7)	0.6101	6 (5, 7)	6 (4, 7)	0.8628
24–48 h after surgery at rest	1 (0, 2)	2 (1, 4)	<0.0001	1 (0, 2)	2 (1, 4)	<0.0001
24–48 h after surgery at activity	4 (3, 6)	4 (3, 5)	0.8763	4 (3, 6)	4 (3, 5)	0.9087
Preoperative HHS	47.6 (34.0, 62.0)	50.6 (35.0, 63.8)	0.5305	47.8 (34.9, 62.8)	52.7 (37.0, 64.3)	0.1754
Preoperative WOMAC	52 (37, 65.5)	52 (38, 68)	0.7670	51.5 (37, 65)	55 (35, 68)	0.7806
HHS at POD 3 months	83.5 (69.4, 88.0)	86.0 (74.0, 90.0)	0.7206	83.5 (69.4, 88.0)	86.0 (75.7, 91.9)	0.5755
WOMAC at POD 3 months	14 (9, 35)	13 (4, 22)	0.0024	14 (9, 35)	13.5 (4, 22)	0.9519

Median (Q1, Q3). PCA, patient-controlled analgesia; PCA + PNB, patient-controlled analgesia and peripheral nerve block; VAS, visual analogue scale;; WOMAC, Western Ontario and McMaster Universities Osteoarthritis index; HHS, Harris Hip Score; POD, postoperative day.

**Table 3:** Subgroup analysis according to leg lengthening (<10 mm and >10 mm).

	Before Propensity Score Matching			After Propensity Score Matching		
	Length Change < 10 mm (N = 190)	Length Change > 10 mm (N = 163)	*p* Value	Length Change < 10 mm (N = 163)	Length Change > 10 mm (N = 163)	*p* Value
Age (years)	59.5 (46, 67)	62 (53, 69)	0.1981	61 (53, 69)	62 (53, 69)	0.8006
Female, *n* (%)	111 (58.4%)	89 (54.6%)	0.4703	87 (53.4%)	89 (54.6%)	0.6276
BMI (kg/m^2^)	24.5 (22.2, 26.7)	24.5 (22.2, 26.9)	0.6743	24.8 (22.5, 27.1)	24.5 (22.2, 26.9)	0.7832
VAS						
0–6 h after surgery at rest	5 (2, 6)	6 (4, 7)	0.0143	5 (2, 7)	6 (4, 7)	0.0378
0–6 h after surgery at activity	7 (6, 8)	7 (6, 8)	0.7299	7 (6, 8)	7 (6, 8)	0.3929
6–24 h after surgery at rest	3 (1, 5)	3 (2, 5)	0.6710	3 (1, 5)	3 (2, 5)	0.1986
6–24 h after surgery at activity	6 (4, 7)	6 (5, 7)	0.8019	6 (4, 7)	6 (5, 7)	0.5381
24–48 h after surgery at rest	2 (0, 3)	2 (0, 3)	0.8506	2 (0, 3)	2 (0, 3)	0.3418
24–48 h after surgery at activity	4 (3, 5)	5 (3, 6)	0.3238	4 (3, 6)	5 (3, 6)	0.2950
Preoperative HHS	51.3 (35.6, 63.7)	48.1 (32.9, 62.7)	0.3753	49.5 (35.6, 63.8)	48.1 (32.9, 62.7)	0.5939
Preoperative WOMAC	51 (36, 65)	53 (37, 68)	0.2678	52 (38, 64)	53 (37, 68)	0.3068
HHS at POD 3 months	86.0 (74.0, 90.0)	84.0 (56.2, 88.0)	0.2593	86.0 (74.9, 90.0)	84.0 (56.2, 88.0)	0.4802
WOMAC at POD 3 months	13 (6, 26)	13 (9, 31)	0.7126	14 (7, 26)	13 (9, 31)	0.8192

Median (Q1, Q3). VAS, visual analogue scale; BMI, body mass index; HHS, Harrison hip score; WOMAC, Western Ontario and McMaster Universities Osteoarthritis index; POD, postoperative day.

**Table 4:** Comparison of clinical outcomes of the 190 patients with leg lengthening <10 mm after surgery.

	Before Propensity Score Matching			After Propensity Score Matching		
	PCA + PNB Group (N = 70)	PCA Group (N = 120)	*p*	PCA + PNB Group (N = 42)	PCA Group (N = 42)	*p*
VAS 0–6 h after surgery at rest	4 (2, 6)	6 (3, 7)	0.0001	5 (2, 6)	6 (4, 7)	0.1580
VAS 0–6 h after surgery at activity	7 (5, 8)	8 (6, 8)	0.0173	7 (6, 8)	8 (5, 9)	0.4382
VAS 6–24 h after surgery at rest	2 (0, 4)	3 (2, 5)	0.0032	2 (0. 4)	3 (2, 5)	0.0165
VAS 6–24 h after surgery at activity	6 (4, 7)	6 (4, 7)	0.3484	6 (4, 6)	6 (5, 8)	0.1122
VAS 24–48 h after surgery at rest	1 (0, 2)	2 (2, 4)	<0.0001	1 (0, 2)	2 (2, 4)	0.0015
VAS 24–48 h after surgery at activity	4 (3, 6)	4 (3, 5)	0.9655	3 (3, 5)	4 (3, 5)	0.6477

Median (Q1, Q3). VAS, visual analogue scale; PCA, patient-controlled analgesia; PCA + PNB, patient-controlled analgesia and peripheral nerve block.

**Table 5:** Comparison of clinical outcomes of the 163 patients with leg lengthening > 10 mm after surgery.

	Before Propensity Score Matching			Before Propensity Score Matching		
	PCA + PNB Group (N = 66)	PCA Group (N = 97)	*p* Value	PCA + PNB Group (N = 26)	PCA Group (N = 26)	*p* Value
VAS 0–6 h after surgery at rest	5 (2, 6)	6 (4, 8)	0.0016	5 (1, 5)	5 (2, 8)	0.4749
VAS 0–6 h after surgery at activity	7 (6, 8)	8 (6, 8)	0.3912	7 (6, 8)	8 (5, 8)	0.8666
VAS 6–24 h after surgery at rest	3 (0, 4)	3 (2, 5)	0.0451	3 (0, 4)	4 (0, 5)	0.4747
VAS 6–24 h after surgery at activity	6 (5, 7)	6 (4, 7)	0.7993	6 (5, 6)	6 (5, 8)	0.0558
VAS 24–48 h after surgery at rest	2 (0, 2)	2 (0, 4)	0.0057	2 (0, 2)	2 (0, 3)	0.4133
VAS 24–48 h after surgery at activity	4 (3, 6)	5 (3, 5)	0.8303	4 (3, 6)	5 (3, 6)	0.3891

Median (Q1, Q3). VAS, visual analogue scale; PCA, patient-controlled analgesia; PCA + PNB, patient-controlled analgesia and peripheral nerve block.

## Data Availability

The data of this study are available from the corresponding author on reasonable request.
